# Suicidality associated with betahistine: A rare case report and systematic review of histaminergic drug-induced depression

**DOI:** 10.1097/MD.0000000000049339

**Published:** 2026-06-19

**Authors:** Jiong Tang, Yuqi Zeng, Xiaoli Jiang

**Affiliations:** a Department of Pharmacy, Chengdu Seventh People’s Hospital (Affiliated Cancer Hospital of Chengdu Medical College), Chengdu, Sichuan, China.

**Keywords:** betahistine, case report, drug-induced suicidality, histamine receptors, pharmacovigilance

## Abstract

**Rationale:**

Drug-induced suicidality is a severe and frequently overlooked area within pharmacovigilance. While the traditional focus has centered on psychotropic medications, non-psychotropic drugs acting on histamine receptors may also induce depression and suicidality through central nervous system effects.

**Patient concerns:**

A 33-year-old male presented with a chief complaint of “dizziness for 1 week.” His dizziness improved after taking a betahistine hydrochloride oral solution, but he subsequently developed symptoms including low mood, emotional detachment, and suicidal ideation.

**Diagnoses:**

Given the close temporal relationship between drug intake and symptom onset, rapid improvement after withdrawal, and assessment by the Naranjo scale and World Health Organization–Uppsala Monitoring Centre system, the depressive symptoms with suicidality were preliminarily judged to be “probable” betahistine-associated adverse reactions (grade 1 [mild]).

**Interventions:**

Betahistine was discontinued immediately and replaced with difenidol hydrochloride tablets to manage the dizziness.

**Outcomes:**

Suicidal ideation resolved completely within 2 days of betahistine discontinuation. No recurrence of psychiatric symptoms was reported during the 3-month follow-up period.

**Lessons:**

This case emphasizes the need for clinicians and pharmacists to move beyond the cognitive bias that only psychotropic drugs carry a significant suicide risk. Vigilance for potential neuropsychiatric adverse reactions, such as depression and suicidality, should be maintained for all drug classes, particularly for non-psychotropic agents acting on histamine receptors. Timely recognition and drug discontinuation are crucial to ensure patient safety.

## 1. Introduction

Medication safety is a cornerstone of global public health, as adverse drug reactions (ADRs) cause significant morbidity and mortality. Drug-induced psychiatric disorders are particularly complex and insidious, and they constitute an increasingly recognized field of pharmacovigilance. Among these, drug-induced suicidality (DIS), which encompasses suicidal ideation, attempts, and completed suicide, is one of the most severe manifestations. Establishing causality between drug use and suicidality remains a persistent challenge in pharmacovigilance research.^[1]^

Historically, DIS research and regulation have concentrated predominantly on psychotropic medications such as antidepressants, antipsychotics, and anticonvulsants.^[2–4]^ Based on trial data and post-marketing reports, the Food and Drug Administration has issued suicidality warnings for several drugs, including black-box warnings. These actions have significantly raised awareness of such risks among clinicians and the public. However, this heightened focus may also foster a bias whereby significant suicide risk is exclusive to psychotropic drugs. In reality, DIS extends beyond psychotropic drugs. A growing body of evidence from case reports and studies indicates that a variety of widely used non-psychotropic drugs may, through diverse pharmacological mechanisms, affect the central nervous system and subsequently induce depression, anxiety, and even suicidal thoughts and behaviors. These drugs span multiple therapeutic areas, including cardiovascular, endocrine, anti-infective, and immunomodulatory. Therefore, identifying the unintended suicide risk associated with non-psychotropic medications is of substantial clinical and societal importance.

Notably, associations with the DIS are often gradually identified after marketing through spontaneous reporting systems and case reports. As these events are rare and confounded by comorbidities or limited awareness, causality is often difficult to establish. This challenge is especially acute for drugs whose labels do not mention this risk, making them more likely to be overlooked with potentially tragic outcomes. A careful review of clinical evidence is essential to update medical understanding, guide prescriptions, and improve drug safety monitoring.

This study was prompted by a clinical case of betahistine-associated suicidality, followed by an extensive literature search for case reports of depression or suicidality associated with histamine receptor-active drugs. The literature search was conducted mainly in PubMed and Web of Science from database inception to the present (i.e., up to 2026), without any lower date restrictions. After rigorous screening, we extracted key information, including demographics, drug regimens, depressive/suicidal manifestations, and outcomes. This study aimed to raise awareness of suicidality and depression induced by non-psychotropic drugs, promote more comprehensive pharmacovigilance, and ultimately safeguard patient medication safety.

## 2. Case presentation

A 33-year-old male (height: 172 cm, weight: 64 kg) presented to a local hospital with a chief complaint of “dizziness for 1 week.” Physical examination revealed the following: body temperature 36.5°C, heart rate 76 beats/min, respiratory rate 20 breaths/min, and blood pressure 135/84 mm Hg. Auxiliary examinations (cranial/cervical computed tomography, transcranial doppler, electronystagmography, and balance tests) revealed no abnormalities. The patient had no history of smoking, alcohol use, or notable family history. He was previously healthy and denied a history of hypertension, hepatitis, tuberculosis, neuropsychiatric disorders, or drug allergies. Consequently, the local hospital’s preliminary diagnosis was “dizziness, etiology undetermined,” and betahistine oral solution (10 mL orally twice daily) was prescribed.

The patient adhered to the medication for 2 days; his dizziness improved, and he subsequently discontinued it. However, five days later, dizziness recurred without an obvious precipitating factor, prompting him to resume betahistine therapy. Nevertheless, after 2 days of resuming therapy, although his dizziness was alleviated, he developed suspected suicidality as an adverse effect, manifesting as low mood, emotional detachment, and suicidal ideation. He promptly consulted a clinical pharmacist and reported similar feelings after the first 2-day course. The pharmacist noted that the patient was a young male with no neurological or psychiatric history. Combined with the results of admission examinations, suicidal tendency-related depressive manifestations caused by genetic, dietary, or environmental factors were excluded. The oral betahistine hydrochloride solution used was a qualified drug product, and its administration complied with the dosage and usage specified in the package insert. The pharmacist concluded that betahistine could not be excluded as the cause, recommended immediate withdrawal, and suggested difenidol as an alternative if required for dizziness. According to the 2021 emergency vertigo guidelines, difenidol is recommended as a short-term vestibular suppressant (≤72 hours) for acute vertigo episodes. Its mechanism of action – muscarinic acetylcholine receptor antagonism – differs from that of betahistine, supporting its use as an alternative. Non-pharmacological options (e.g., vestibular rehabilitation) are available for long-term management but are not indicated for this acute setting.

Two days later, telephone follow-up confirmed that the suicidal ideation had not recurred after the discontinuation of betahistine. The patient was subsequently monitored for 3 months, during which time he reported no recurrence of suicidality, emotional detachment, or depressive symptoms (Table S1, Supplemental Digital Content 1).

Two standardized ADR assessment tools were used to evaluate the causality between betahistine use and depressive symptoms and suicidality. According to the Naranjo Adverse Drug Reaction Probability Scale (scores: definite >9; probable 5–8; possible 1–4; doubtful ≤0),^[5]^ a score of 6 was obtained, indicating a “probable” causal relationship. Concurrently, the World Health Organization–Uppsala Monitoring Centre (WHO–UMC) causality assessment system was applied, yielding a classification of “Probable” for the association between betahistine and the development of suicidality. Based on these 2 independent assessments, the relationship between betahistine use and the patient’s depressive symptoms was preliminarily determined to be “probable.” Several pieces of evidence support this causality assessment. First, alternative etiologies were systematically ruled out: the patient had no personal or family history of psychiatric disorders, no recent psychosocial stressors, normal neuroimaging and vestibular tests, no concomitant medications known to induce depression or suicidality, no alcohol or substance abuse, and no unusual changes in dietary patterns or composition that could potentially affect monoamine oxidase activity. Second, key temporal features strongly favor a drug-induced reaction: a close temporal sequence between betahistine administration and symptom onset on 2 separate occasions; complete symptom resolution within 48 hours after withdrawal (positive dechallenge); and symptom recurrence upon rechallenge, which effectively excludes most confounding variables such as disease progression or psychological stress. Particularly, a positive rechallenge is a rare but highly compelling pharmacovigilance finding. Taken together, these findings support a “probable” causal association of betahistine with depressive symptoms and suicidality. In addition, because the patient’s suicidality was mild (resolved 2 days after withdrawal) and did not affect daily living, it was graded as grade 1 according to the Common Terminology Criteria for Adverse Events. Generally, for DIS of grade 1 to 2 severity, gradual dose reduction or discontinuation is sufficient. Management of severe reactions (grade 3 or higher) may require pharmacological and/or psychotherapeutic interventions.

## 3. Discussion

Betahistine was approved for marketing in Canada in 1968,^[6]^ and has since been employed in more than 80 countries, with an estimated patient exposure exceeding 100 million. It is commonly prescribed for vertigo or dizziness due to Meniere’s disease and vestibular dysfunction.^[7,8]^ Common adverse reactions documented in the prescribing information included dry mouth, loss of appetite, palpitations, and exacerbation of peptic ulcers. Additional adverse effects include sweating, hemorrhagic cystitis, fever, sinus tachycardia, edema, and hypotension. A comprehensive literature search using keywords, including “betahistine hydrochloride,” “betahistine mesylate,” “suicidal ideation,” and “suicide attempt,” suicide attempts, across major domestic and international databases yielded no prior clinical reports linking betahistine to suicidality. A supplementary search using “psychiatric disorders” identified 1 study on behavioral and psychiatric symptoms in cognitive neurology that observed associations between dysthymia and betahistine when standard treatments were excluded.^[9]^ Furthermore, a retrospective analysis of betahistine safety issues reported neuropsychiatric ADRs, including insomnia, irritability, and agitation, suggesting an impact on the neuropsychiatric system.^[6]^ A study using the FEARS database identified betahistine use as a risk factor for suicide attempts.^[10]^ In addition, a search of the WHO VigiAccess database using “betahistine” revealed nearly 16,000 adverse event reports, including 10 cases of suicidality and suicide attempts. Collectively, the existing literature supports a potential association between betahistine and suicidality, although no individual case reports have been previously documented. To our knowledge, this is the first documented case suggesting a potential association between betahistine use and the onset of suicidality.

Betahistine, an histamine H3 receptor antagonist, increases histamine release and promotes wakefulness. Emerging evidence suggests a significant association between hyperarousal and depression. The neurobiological basis for this link lies in the dysregulation of the brain’s arousal system in depressive disorders. Studies have demonstrated that patients with depression exhibit upregulated cerebral arousal, which is considered a pathophysiological mechanism underlying sleep disturbances, inner tension, autonomic hyperarousal, and anhedonia.^[11]^ Neuroimaging has shown that during non-rapid eye movement sleep, patients with depression have persistently elevated metabolism and regional blood flow in multiple brain regions, including the limbic system, anterior cingulate cortex, thalamus, and basal ganglia.^[12]^ The potential role of betahistine-induced histaminergic modulation in disrupting the arousal balance warrants further investigation as a possible mechanism contributing to depressive symptoms in susceptible individuals.

However, the precise pharmacological mechanism by which betahistine induces suicidal ideation remains elusive and complex. Betahistine acts as a weak H1 agonist and a strong presynaptic H3 antagonist; this dual action underlies its efficacy in improving vertigo and cerebral blood flow. Notably, the histamine H3 receptor functions not only as an autoreceptor, providing negative feedback on histamine synthesis and release, but also as a heteroreceptor, modulating the release of other key neurotransmitters in the brain, including norepinephrine, serotonin, and γ-aminobutyric acid.^[13,14]^ Dysregulation of the function and homeostasis of these neurotransmitter systems is closely associated with the development of depression. Normal brain function relies on a precise balance maintained by the complex interaction between excitatory (e.g., glutamate) and inhibitory neurotransmitters (e.g., γ-aminobutyric acid).^[15]^ We hypothesize that the adverse effects stem not from a simple linear pathway, but rather from a disruption of the intricate neurochemical balance within the limbic system. Histaminergic neurons project extensively throughout the brain and serve as key modulators of excitatory–inhibitory balance. Betahistine may indirectly disrupt this excitatory–inhibitory balance within critical mood-regulating circuits, thereby precipitating depression-like behaviors. In summary, suicidality as a manifestation of clinical depression may be linked to betahistine-induced disruption of neurotransmitter homeostasis via histamine receptors, leading to complex neural regulatory effects.

More importantly, the temporal features strongly suggest patient-specific idiosyncratic reactions. The patient experienced mild suicidality during the initial treatment period and more severe symptoms upon rechallenge, indicating reproducible biological sensitivity. Betahistine is safe for millions but causes severe symptoms in this patient, underscoring individual vulnerability. We hypothesized that this vulnerability may stem from the following: pharmacogenomic factors such as unknown polymorphisms in the histamine receptor genes or metabolic enzyme genes. Consistent with findings in pharmacogenomic studies in which specific gene polymorphisms (e.g., 5-HT2A receptor and CYP2D6) predict susceptibility to ADRs, this patient may harbor unknown genetic variants leading to an idiosyncratic pharmacodynamic response.^[16]^ preexisting subclinical network instability in mood-regulating circuits. Betahistine may have acted as a “second hit,” destabilizing these circuits and unmasking a latent depressive phenotype. This case highlights that even medications with an “activating” pharmacological profile can trigger paradoxical and severe neuropsychiatric events in vulnerable individuals. This underscores the need for personalized pharmacovigilance.

Several studies have suggested an association between histaminergic drugs and depression. A 2025 large-scale pharmacovigilance study identified signals for “various depressive manifestations” associated with drugs such as cetirizine, promethazine, and diphenhydramine in adolescents and children.^[17]^ Similarly, an analysis of safety signals from the WHO global database up to October 2017 documented 49 case reports linking desloratadine to depression. Some cases resolved with withdrawal and recurrence upon rechallenge, suggesting causality.^[18]^

We conducted a systematic literature search for case reports on depressive manifestations associated with histamine receptor-active drugs. This search utilized keywords including “histamine,” “depression,” and “case report” across major international and domestic databases. Apart from nonselective agonists (e.g., histamine hydrochloride) used in specific diagnostics or rare diseases and betahistine, few common drugs directly activate histamine receptors. To date, no case reports on depression-related ADRs have been documented for agonist-type drugs. In contrast, case series have consistently reported that H1/H2 antagonists (e.g., cetirizine and cimetidine) can induce reversible depressive syndromes within days to months (Table S2, Supplemental Digital Content 2). Common characteristics across these cases include: close temporal link; resolution after withdrawal and recurrence on rechallenge; age was not a determining factor; and Naranjo scores “probable” to “definite.” Notably, approximately half of these cases involve suicidal ideation or behavior, suggesting that histaminergic imbalance may elevate suicide risk. Therefore, for any histamine receptor-active drug, newly emergent symptoms, such as depressed mood, loss of interest, or suicidal ideation, should be considered as potential adverse reactions. Prompt drug discontinuation, suicide risk assessment, and if necessary, alternative therapies and psychological interventions are warranted.

We acknowledge the limitations of a single case report, including the inability to establish definitive causality, potential recall bias, and lack of generalizability. Isolated case observations cannot quantify risk or rule out confounders such as comorbidities or concomitant drugs. Thus, large-scale pharmacovigilance analyses and other real-world data are urgently needed to systematically evaluate the association of betahistine (and other histamine receptor-modulating drugs) with suicidality. Such analyses would provide robust signal detection, estimate incidence rates, adjust for confounders, and complement the hypothesis-generating role of case reports.

## 4. Conclusion

Based on clinical observations, this article presents the first reported case linking betahistine use with depressive symptoms and suicidality. Furthermore, a systematic review of the existing case literature delineated a potential association between histamine receptor-active drugs and the risk of depression or suicidality. This finding highlights a long-standing yet often overlooked “blind spot” in drug safety. This report aims to alert clinicians and pharmacists to break through the cognitive limitations of “only psychotropic drugs carry suicide risk,” strengthen the monitoring and identification of neuropsychiatric adverse reactions induced by various medications, and prompt intervention, including discontinuation of the suspected agent, is crucial for improving medication safety and mitigating potential risks. Future research should focus on quantifying these risks through large-scale epidemiological studies and elucidating the underlying biological mechanisms to better inform clinical practice and drug safety regulations.

## Author contributions

**Conceptualization:** Jiong Tang, Xiaoli Jiang.

**Data curation:** Jiong Tang, Yuqi Zeng, Xiaoli Jiang.

**Formal analysis:** Jiong Tang, Xiaoli Jiang.

**Writing – review & editing:** Jiong Tang.

**Writing – original draft:** Xiaoli Jiang.




