# Supplementary material for: Suicidality associated with betahistine: A rare case report and systematic review of histaminergic drug-induced depression
Source: Medicine (Baltimore). 2026 Jun 19;105(25):e49339. doi: 10.1097/MD.0000000000049339 (PMC13286311; doi:10.1097/MD.0000000000049339)
Supplement: Supplementary file 1 [file medi-105-e49339-s001.docx]

Supplementary Table 1. Timeline of key clinical events

| Day | Event | Clinical status | Intervention / Outcome |
| --- | --- | --- | --- |
| 1–2 | First betahistine course (10 mL orally twice daily) | Dizziness improved | – |
| 3–7 | Drug-free interval | No dizziness initially; dizziness recurred on day 7 | – |
| 8–9 | Second betahistine course | Dizziness relieved, but low mood and emotional detachment emerged on day 9 | – |
| 9 | Suicidal ideation developed | Mild suicidality (CTCAE Grade 1) | Betahistine immediately discontinued |
| 10 – 11 | Post-withdrawal period | Suicidal ideation persisted transiently | Difenidol prescribed as alternative for dizziness |
| 12 | Complete symptom resolution | Suicidal ideation resolved entirely | Patient recovered without psychiatric intervention |
| 12 – 3 months | Follow-up period | No recurrence of depression or suicidality | Confirmed by telephone follow-up and outpatient visit |
