# Supplementary material for: Suicidality associated with betahistine: A rare case report and systematic review of histaminergic drug-induced depression
Source: Medicine (Baltimore). 2026 Jun 19;105(25):e49339. doi: 10.1097/MD.0000000000049339 (PMC13286311; doi:10.1097/MD.0000000000049339)
Supplement: Supplementary file 2 [file medi-105-e49339-s002.docx]

Supplementary Table 2. Case report on histaminergic drug-induced depression

| **Drug classification** | **Main indications** | **Drug name** | **Age/**  **Gender** | **Concomitant disease/past medical history** | **Case description** | **Prognosis of disease/Outcome** | **Naranjo evaluation scale** | **Authors (year)** |
| --- | --- | --- | --- | --- | --- | --- | --- | --- |
| Histamine H1 Receptor Antagonists | allergic rhinitis, urticaria | Cetirizine | 18/F | No psychiatric history, no substance misuse, no family history of mental illness | After starting cetirizine 10 mg daily for allergic rhinitis, developed persistent delusions (being raped, demon-possessed), marked depression and suicidal ideation within 1 week; symptoms lasted 3 months and intensified | Delusions and suicidal ideation resolved within 4 days of discontinuation; euthymic mood restored; discharged after 8 days, remained symptom-free on outpatient follow-up | Probable | Garden BC, *et al.* 2013^[1]^ |
|  |  | Levocetirizine | 9/M | Previously healthy, no psychiatric history, no concomitant drugs or herbals | Oral levocetirizine 5 mg/day started for allergic rhinitis; on day 9 developed severe obsessive-depressive thoughts, mixed anxiety-depression, excessive crying, low mood, anhedonia; symptoms persisted for another 7 days while drug continued | Symptoms completely resolved ≈12 days after discontinuation; no recurrence on follow-up | Probable | Carnovale C, *et al*. 2017^[2]^ |
| Histamine H2 Receptor Antagonists | GERD, peptic ulcer | Ranitidine | 45/F | No personal/family history of depression, also on prednisone & diazepam | 4 weeks after starting ranitidine 150 mg bid developed anxiety, depression, anhedonia, and so on | All symptoms remitted within 2 weeks; remained well at 1-year follow-up | Probable | Billings RF, *et al*. 1986^[3]^ |
|  |  |  | 78/M | 8 weeks post-MI, also on furosemide, isosorbide dinitrate, digoxin, oxazepam | ~5 weeks after ranitidine 150 mg bid developed depression, irritability, and so on | Stopped ranitidine; improvement within 1 week, much better at 3 weeks; no recurrence over 6 months | Probable | Billings RF, *et* *al*. 1986^[3]^ |
|  |  |  | 65/M | On ranitidine 6 months for “dyspepsia”, also on digoxin | During therapy developed 4-month depressed mood, anorexia, psychomotor retardation and others; symptoms improved after self stopping medication for 2 weeks; symptoms recurred on rechallenge | Abruptly stopped ranitidine; “100 % better” within 6 days, depressive & cognitive symptoms fully resolved | Almost certain | Billings RF, *et al*. 1986^[3]^ |
|  |  | Cimetidine | 40/F | Previously healthy, no psychiatric history, receiving monthly estrogen injections only | Start treating peptic ulcers with 300mg of cimetidine once a day; Significant depression and complete loss of sexual desire appeared 3 days later, worsened within 4 weeks, and even contemplate suicide | Within 24 h of discontinuation libido returned and depressive symptoms fully resolved | Almost certain | Pierce JR Jr. 1983^[4]^ |
|  |  |  | 35/F | Hypothyroidism on stable thyroxine, no personal/family history of affective disorder | Due to oral administration of cimetidine (300 mg qid) for reflux esophagitis, fatigue, loss of appetite/weight, drowsiness, lack of interest, etc. appeared 2 weeks later, and suicidal ideation occurred 1 week before admission | Cimetidine stopped on admission; improved in 10 days. Mild residual depression 1 week post-discharge → imipramine 150 mg; euthymic in 2 weeks, maintained on 75 mg without relapse | Probable | Billings RF, *et al.* 1981^[5]^ |
|  |  |  | 67/M | Past prostatectomy, asthma, no family history of mood disorder | Cimetidine 300 mg qid for peptic ulcer; within 1 month lethargy, then over 11 months progressive fatigue, anhedonia, suicidal rumination, others | Cimetidine stopped, imipramine 75 mg started; discharged 3 weeks later. Depression recurred on tapering, stable on long-term 75 mg/d | Probable | Billings RF, *et al.* 1981^[5]^ |
|  |  |  | 81/M | No prior psychiatric history, long-term cimetidine for recurrent peptic ulcer | Over several months became “disgusted with life,” poor concentration, lost interest/pleasure, others; on cimetidine 300 mg qid for months | Within 2 days of discontinuation became optimistic; discharged cheerful | Almost certain | Jefferson JW.1979^[6]^ |
|  |  |  | 48/M | past medical history of recurrent peptic ulcer only, otherwise no significant physical or psychiatric illness | Started cimetidine 300 mg q.i.d. 7 months prior; over 7 months developed marked depression—sadness, worthlessness, loss of interest, disappointment and so on; unable to work and suicidal pre-admission | Ceasing cimetidine significantly improved depression after 2 days; After challenging medication again, depression recurred, and symptoms quickly improved after discontinuing the medication again | Almost certain | Crowder MK, *et al.*1980^[7]^ |

GERD: gastroesophageal reflux disease; F: female; M: male.
